# Indents

**Published:** 1922-03

**Authors:** 


### INDENTS

**☞Brief Articles of Technical or Scientific Value will receive notice and comment. Contributions invited.**

---

COSTS AND RECEIPTS IN PRACTICE. In the first article of this series, practices were grouped by the amounts of gross annual receipts, which is a common but not very satisfactory method of arrangement. 176 practices reported annual gross receipts of less than $5,000. The average net receipts were $2,463, but this average does not give a correct picture of the conditions within the class.

Four of these practices reported office expenses of 55 per cent. and annual net receipts of less than $1,000.

Twelve practices reported net receipts between $1,000 and $1,500. One dentist who conducts a practice of $4,544 gross had left for remuneration not more than $1,260.

Twenty-seven practices reported between $1,500 and $2,000 annual net receipts. The gross receipts ranged from $2,300 to practically $4,800. The $2,300 practice returned $1,550 net, while the $4,800 practice returned only $1,930 net.

Forty-five practices showed net annual receipts between $2,000 and $2,500. The annual gross receipts ranged from $2,400 to $4,800. The $2,400 practice showed nearly as large annual net returns as the $4,800 practice.

Fifty-three practices showed net annual receipts between $2,500 and $3,000. The annual gross receipts ranged from $3,200 to $4,900. The $3,200 practice showed net returns practically identical with those of the $4,900 practice.

Twenty-five practices showed net annual receipts between $3,000 and $3,500. The gross receipts ranged from $3,800 to practically $4,900. The net receipts in the $3,800 practice were practically identical with those of the $4,800 practice.

Ten practices showed net receipts between $3,500 and $4,000. If the reports are accurate, the practices are well conducted from the economic point of view, though it is doubtful if a dentist can conduct his practice on an expenditure of only 15 per cent. of the gross receipts.—George Wood Clapp, in *Dental Digest.*

---

CHEVALIER OF THE LEGION OF HONOR—DR. HERBERT L. WHEELER. The Republic of France is ever appreciative of the efforts of those whose public spirit leads them to accomplish valuable work in the hour of need.

Dr. Herbert L. Wheeler, of New York, gave his time, knowledge and energy to the dental reconstruction of the French soldiers during the war. In recognition of the value of his work and the good he accomplished, France has bestowed upon Dr. Wheeler the Order of Chevalier of the Legion of Honor.

This is not only a great honor to Dr. Wheeler, but is an honor to the entire dental profession.

The great men of the world have been proud to wear the blood-red ribbon of the Legion of Honor.

Dr. Wheeler, we congratulate you!—*Oral Hygiene.*

---

THE SUCKER LIST. After everybody else in the country has been tried out on a stock jobbing proposition, the dentist is approached as a last resort.

There are two ways to look at this phenomenon: either the dentist is considered an easy mark to clean up on at the last, or else he is so hard-boiled that the agent gets all of the experience he can before tackling the dentist.

When you make an investment, buy something that you can get your money out of.

Business profits are made very largely upon the shifting basis; that is, the money changes hands and what you lose is the other fellow's profit.

Why should you work and save in order to help a stock-selling shark make a record?

When you buy stocks and bonds, do as you expect your patients to do.

Your patients get very little sympathy from you if they take the advice of a quack.

You have taught them to trust an expert.

Do the same thing. Ask your banker what to buy.

He knows—or he wouldn't have a bank.—*Oral Hygiene.*

---

AGAIN, NO SHORTAGE OF PHYSICIANS. On another page we publish a report [1] on the scarcity of physicians in the rural districts of Pennsylvania. It shows that there is an overlarge supply of physicians in the cities of that state which more than offsets the lack of physicians in rural districts. This further corroborates the statements repeatedly made by *The Journal*, that, although physicians are scarce in rural communities, there is no scarcity of physicians in the country as a whole; the conditions referred to are found not only in Pennsylvania but in all the other states as well. The latest figures [2] show that Pennsylvania is well supplied with physicians as compared with the rest of the country. It has one physician to every 768 people, while in the country as a whole there is one to every 726 people. This report makes no reference to the real reason why physicians are not in rural communities. In a conference in Kentucky held recently between the House of Delegates of the Kentucky State Medical Association and members of the legislature, this problem was the subject of a practical discussion. There it was clearly demonstrated that the lack of doctors in rural communities was not due to a general scarcity of physicians, nor to higher educational

---

1 Report of the Committee on Medical Education of the Philadelphia County Medical Society, p. 453.

2 See table on Proportion of Physicians to Population, J. A. M. A. 76: 1248 (April 30), 1921.

requirements, but to economic conditions—to the fact that physicians will not locate in districts where they can not secure a reasonable income and where living conditions are poor. The saving of one or two years of time in intermediate and secondary education, as recommended in the Pennsylvania report, is indeed desirable; but that reform would have no effect whatever in supplying doctors for rural communities. It is quite clear that the only way by which physicians can be induced to locate in rural districts is to make those districts more attractive places in which to live, from the professional, social and economic points of view.—*Journal A. M. A.*

---

INCLUSIVE AND DISCRIMINATING DIAGNOSIS. The question of diagnosis is the real basic problem in the practice of medicine and dentistry, and yet we are prone to forget this important fact simply because our patients come to us asking primarily for treatment. We, in turn, desire to give immediate relief and center our attention upon the means for doing this. Thus the question of diagnosis is forced into the background or taken up as a secondary consideration. This is fundamentally wrong. The scientific practice of dentistry demands a complete and intelligent review of all cases for the purpose of establishing diagnostic conclusions. In practicing this principle we not only avoid those disconcerting mistakes, such as the essayist mentioned, but also have the satisfaction of obtaining real results from subsequent therapy and surgery. Therefore with all the arguments advanced in favor of the use of roentgenology as routine practice in dentistry and oral surgery I am most heartily in accord, and little has been left unsaid on this side of the question.

As I can not disagree with the essayist, I believe the time may be profitably used to emphasize somewhat more in detail one of the points that the writer merely mentioned. Early in his paper, Dr. Thoma said, "Roentgenology should not be allowed to replace all other methods of diagnosis. It should be used in conjunction with clinical examination." This, I believe, lays bare the one possible harm that may creep into our practices as a result of the introduction of roentgenology. So strongly do I feel about this that I almost dare to say there is more danger of superficial work in the diagnostic field since the advent of roentgenology than there was previous to it. It is the natural reaction following the universal adoption of this wonderful aid to methods of diagnosis. The radiogram has revealed so much about many cases that formerly were quite obscure that we rush to it as a panacea for all problems and in so doing neglect to make a careful and thorough study of the clinical symptoms presented by the case. Consequently we fail to place ourselves in a sufficiently enlightened condition to reach a conclusive diagnosis. It is impossible to interpret a film with complete intelligence unless one knows intimately all of the clinical data associated with the case. Hence it is of extreme importance that we train ourselves to carefully weigh the deductions obtained from the older methods of case study and formulate a tentative diagnosis before consulting the visualized evidence.

I believe the radiogram should be the last court of inquiry and that we should approach and use the data recorded thereon as a means of confirming our conclusions instead of first consulting the film with the idea of establishing a diagnosis. Anyone who routinely studies his cases in this manner will find himself becoming more and more careful in his clinical examinations, for unconsciously he is anxious to arrive at a conclusion that will be consistent with the accurate record found upon the roentgenogram, so that the problem takes the form of a contest between the operator's power of observation and deduction and the unerring revelations obtained from the use of a scientific machine. It is also well to remember that there are many cases presenting lesions that are only apparently associated with the hard structures and these will produce a radiogram with negative findings. This is somewhat disconcerting to the man who has allowed himself to depend entirely upon the radiogram for diagnosis. For this reason we must keep in constant training all of the facilities available for case study so that we may be fully prepared for just such an emergency. The usual case that presents itself can be diagnosed by anyone, for it offers symptoms and signs of such characteristic hue that they are quite evident to even the most superficial observer. On the other hand, the obscure case can only be unraveled by piecing together the broken threads that are gathered here and there from all the sources at our command, included among which are the wonderful revelations of the roentgenogram. By all means let us adopt roentgenology as a routine procedure in our practices, but let us not allow it to displace or exclude a single one of the older methods of clinical study that have proven themselves so reliable and invaluable in our efforts to establish accurate diagnosis. —R. H. W. Strang, in *Cosmos,* February, 1922.

---

CONCERNING THE ETIOLOGY OF PYORRHEA ALVEOLARIS. The etiology of pyorrhea alveolaris has probably received more attention and has been discussed at greater lengths by dental investigators than any other subject in the field of dental pathology. The Index of the Periodical Dental Literature for the years from 1911 to 1915 records several hundred such contributions. Dental research workers have studied this pathologic complex from every angle, and in recent years the investigations on the patho-histology of the disease and its bacterial exciters have further added to a voluminous literature. Whether the complex is of systemic or local origin, or a combination of both, seems to have received a good share of the time and energy devoted to the solving of this dental problem, and it is regrettable to contemplate that, notwithstanding all the efforts so far expended, no solution has been so far offered which has received unanimous acceptance. The laboratory or the clinic, each taken as a separate method of investigation of this or any other pathologic entity, affords at best but incomplete evidence as to the cause or causes responsible for the chain of symptoms to which such a pathologic disturbance may give rise. Laboratory findings must be invariably confirmed by clinical evidence, and the latter must harmonize with the former before framing conclusions worthy of serious consideration.

Contributions on the etiology of pyorrhea alveolaris have appeared recently in the dental journals intended to prove that the initial lesion in so-called pyorrhea alveolaris does not occur in the gingival structures; that the first evidence of retrograde changes—of nutritional order—is detected in the osseous tissue of the alveolar process, the gingival inflammatory reactions occurring subsequent to a loss of integrity in the bony support of the tooth. That this theory is correct in a small number of cases is well established by clinical and laboratory findings; that it does not conform to the facts in the vast majority of instances is likewise attested by the same methods of investigation. To any dentist who has given any thought at all to the etiology of chronic infections in the investing tissues,—who has observed the pathologic complications in the investing tissues of occlusal and restorative faults, who has seen the staggering prevalence of gingival and peridental disease traceable to faulty dental reparative operations,—to him it would be difficult to accept the theory that pyorrhea alveolaris, in the largest percentage of cases, is due, in the very beginning, to causes other than the involvement of the gingival tissues.

The theory of initial osseous involvement explains the changes which occur in certain cases of pyorrhea alveolaris in which, to all appearances, no local cause or group of causes can be found to explain the retrograde changes in the investing tissues; these are the cases associated with circulatory disturbances, degenerations in the vessel walls and endocrine derangements.—Julio Endleman, in *Pacific Dental Gazette.*

---

THE TREATMENT OF INFECTED DENTIN. The disease that most commonly attacks the human teeth is dental caries. When caries has gained entrance through the enamel, its progress is generally quite rapid and many times before it is drawn to the attention of the dentist the disease has extended almost to, if not actually into, the pulp. As the result of such conditions there are today many devitalized teeth. It has been common practice under such conditions to destroy the pulp and remove it. This was done because dentists believed that the life of the pulp was endangered, for where they had attempted to preserve the life of this organ pain generally followed.

It is our belief that a tooth affected by dental caries where the lesion extends close to the pulp is seriously involved and is in no condition to recover without treatment. To immediately place in or over it a large metal restoration or filling is an improper dental procedure, regardless of any desire to finish up the case. The history of pulps dying and metastatic sequelae inaugurated is widely accepted. The serious nature of the resulting lesion should give us pause in such procedure. All such teeth should be given the proper treatment, and this includes a period of observation before being required to withstand the injury from shock of thermal irritation resulting from large metal fillings.

There are present in the mouth certain conditions and influences detrimental to the tooth pulp and against which it will generally protect itself if vital. In the event of teeth wearing down through attrition in mastication, it will be found that through the activity of the odontoblasts, a formation known as secondary dentin, which occurs coincidentally with the recession of the pulp, takes place. This is nature's method of maintaining a safe amount of tooth structure between the outside surface of the tooth and the pulp, as a protection from pulp exposure. This is also observed in the instances where dental caries develops slowly.

However, in most instances caries advances rapidly and the pulp is unable to protect itself. It is then that infection approaches very closely and sometimes is in actual contact with pulp tissue. In these cases where there may be no actual perforation of dentin into the pulp by caries and where the pulp is still vital and hyperemia or pulpitis has not advanced too far, the operation for preserving the pulp vitality consists as follows: In undertaking the operation for the treatment of infected dentin and maintaining the vitality of the tooth pulp, it must be distinctly borne in mind that this operation does *not lend itself to a careless technic.* The same care employed in inserting a gold foil filling must be used. The rubber dam should be placed around the tooth and all overhanging enamel should be broken away. Very cautiously remove the disintegrated dentin by using *sharp* excavators and burs, cutting the softened dentin in such a direction that the instrument is moving *away* from the pulp instead of toward it. *Keep away from the pulp. It is necessary that this work be done under direct vision with the cavity dry and well illuminated.* Many pulps are needlessly exposed because of the fact that the operator can not see his field of operation. Control any temptation to remove the last layer of discolored dentin which immediately overlies the pulp. Success in this operation seems to be determined very largely by preventing pulp exposure. (The operation here advocated is not a "pulp capping.") It is possible to determine from the radiogram the shape of the pulp chamber. This must be ever kept in mind and the radiogram frequently consulted.

When the disintegrated and softened dentin has been removed but the vital pulp *left unexposed*, a pellet of cotton saturated in a warmed one per cent. Nuklorene (a form of Dakin's solution) is placed in the cavity and allowed to remain for five or ten minutes. Following this the cavity is dried. (Do not use alcohol and warm air.) For the purpose of sterilizing the layer of dentin overlying the pulp, sterident is used. This consists of a powder, magnesium, zinc and silver nitrate, and a liquid, pimento eugenol. Its method of application is as follows: Mix the powder and liquid to a somewhat thick consistency and place a small portion in the base of the cavity. Gently force this to place with a pellet of cotton or a round burnisher. Be careful to trim it away from the margins.

Following this we apply oxychloride of zinc cement. This is used because it is impervious to moisture. It is mixed to a thick, creamy consistency and placed over the sterident. The entire base of the cavity must be covered with the oxychloride of zinc cement (Ames). The balance of the cavity is filled with oxyphosphate cement. (S.S.W. silver cement is used for this purpose.) In some instances where it is necessary to maintain contact with adjacent teeth, a thin veneer of alloy is used.

The tooth is kept in this condition for a period varying from six months to a year, depending on the depth and extent of the cavity, when it is generally safe to place a permanent filling therein. During this time it should be radiographed and tested for vitality. It will be found that these teeth remain in a comfortable condition and retain their vitality. Owing to the danger of losing a pulp, no change in any of the materials used is made once they prove satisfactory. For this reason detailed information in this respect has been given. It is not presented as a cure-all for various diseased conditions of the pulp. It is not a pulp capping. The indications for its use must be left to the intelligent judgment of the dentist. It is offered as a solution of the problem which has been so unsatisfactorily solved in the past by devitalizing teeth.

In children's preventive dentistry, while our aim is to prevent cavities developing in the deciduous teeth, yet it will frequently be found that cavities will have developed before the parent brings the child to the office. In these cases the operation is somewhat modified by leaving out the oxychloride of zinc cement.—Best and Waldron in *N. D. A.*

---

The Michigan State Medical Society and the Medical School of the University of Michigan. *To the Editor:*—During the past years, and especially through this last year, rumors and misstatements have appeared in the medical as well as the lay press. They were concerned with compulsory health insurance, ''state medicine,'' socialization of medicine, administrative policies of the Medical School of the University of Michigan, and the antagonism of the medical profession of Michigan.

January 11, 1922, in Detroit, the council of the Michigan State Medical Society, and President Burton and a committee of the University of Michigan met in conference. As a result of that conference, we desire to make the following announcements to the profession and public of this country over our signatures:

1. A basis of mutual understanding has been reached, and past apparent differences have been obliterated.

2. The university and its medical school are not in favor of ''state medicine,'' so called, nor do they indorse or subscribe to those policies or movements that have for their object the establishment of any such forms for the practice of medicine.

3. Dr. Hugh Cabot, dean of the medical school, has been and is opposed to "state medicine," so called. He desires his opposition to be known to the entire profession, and that in the past he has been unjustly accused of being favorable to that type of socialization of medical practice.

4. In response to the invitations of the president and council of the Michigan Medical Society, the University of Michigan, through its extension division and medical school, has expressed its desire and readiness to educate the public in regard to scientific medicine and the benefits to be derived therefrom.

5. The medical school is concerned chiefly with the education of students in scientific medicine, with the promotion of medical research, and with co-operation with the profession in the advancement of scientific medicine in Michigan.

To these ends have we pledged ourselves and through duly appointed representatives we propose to enter into a campaign of concerted and co-operative activity. Coincident with this action, we believe that the profession at large should be acquainted with our avowed attitude. We therefore issue this statement at this time for the explicit purpose of discrediting false assertions of the past and to make clear for the future the policies and purposes of the principles concerned in this announcement.—The Michigan State Medical Society, W. J. Kay, President, W. J. Du Boise, Chairman of the Council; The University of Michigan, Marion L. Burton, President, Hugh Cabot, Dean of the Medical School.—*Journal N. M. A.*

